# Efficacy of intravenous sedation and oral nifedipine in dental implant patients with preoperative hypertension - a retrospective study of 516 cases

**DOI:** 10.1186/s40729-015-0004-4

**Published:** 2015-03-18

**Authors:** Motoshi Kimura, Yoshihiro Takasugi, Shigeyoshi Hanano, Katsuyuki Terabe, Yuko Kimura

**Affiliations:** 1 Hanano Dental Clinic, 4-2-3 Yamanoue, Hirakata, Osaka 573-0047 Japan; 2 Department of Anesthesiology, Kinki University Faculty of Medicine, 377-2 Ohno-Higashi, Osaka-Sayama, Osaka 589-8511 Japan; 3 Terabe Dental Clinic, 4-249 Sakae-cho, Tsu, Mie 514-0004 Japan; 4 First Department of Internal Medicine, Osaka Medical College, 2-7 Daigaku-machi, Takatsuki, Osaka 569-8686 Japan

**Keywords:** Oral surgery, Dental implant, Hemodynamics, Intravenous sedation, Nifedipine administration

## Abstract

**Background:**

To examine the effects of intravenous sedation and oral nifedipine on blood pressure and pulse rate in patients with perioperative high blood pressure undergoing implant surgery, the clinical records of dental implant patients managed by intravenous sedation at our outpatient dental offices were retrospectively evaluated.

**Methods:**

A total of 516 clinical charts were evaluated. The subjects were divided into two groups: a normotensive group with no history of hypertension and a hypertensive group with a history of hypertension. The patients in the hypertensive group were further divided into two subgroups: with or without nifedipine administration before operation. Systolic blood pressure (SBP), diastolic blood pressure (DBP), pulse rate (PR), and rate pressure product (RPP) were assessed.

**Results:**

In 30 patients (33%) of the hypertensive group, the high blood pressure on arrival obviously declined to around or less than 160 mmHg; in the remaining patients in the group who showed a mean SBP of 182.1 ± 13.8 mmHg on arrival, the blood pressure did not decrease after a 30-min rest. Oral nifedipine administered to the patients with sustained high blood pressure decreased SBP to 144.7 ± 23.1 mmHg in 28.1 ± 9.3 min after administration, comparable to that in hypertensive patients without nifedipine.

**Conclusions:**

For patients with stage 2 hypertension before operation, it may be difficult to maintain the recommended blood pressure during surgery by only intravenous sedation; reduction of blood pressure by antihypertensive drugs may be necessary.

## Background

Osseointegrated dental implants were introduced in Japan in 1983, and the procedures are now performed very frequently. Dental implants are placed in a wide age range of patients, including elderly patients with hypertension. Patients with very high blood pressure are at great risk for acute medical problems when undergoing stressful dental procedures, such as oral surgery, periodontal surgery, and placement of dental implants [1].

Patients with normal blood pressure (<120/80 mmHg), prehypertension (120 to 139/80 to 89 mmHg), or stage I hypertension (140 to 159/90 to 99 mmHg) may receive regular dental care, but those with stage 2 hypertension (≥160/≥100 mmHg) should receive noninvasive treatment only and be referred to the physician for immediate follow-up [2]. Normotensive individuals may develop signs of hypertensive encephalopathy at blood pressures as low as 160/100 mmHg, whereas chronically hypertensive patients can tolerate higher blood pressure and may not do so until the blood pressure rises to 220/110 mmHg or above [3]. Although no recommendation has been presented on the optimal level of blood pressure to avoid hypertensive complications during invasive dental treatments, blood pressure in hypertensive patients should be maintained below 160/100 mmHg.

Pain, stress, or anxiety-related dental procedures can raise blood pressure in both hypertensive and normotensive patients [4]. We have employed intravenous sedation to manage patients with hypertension as well as dental anxiety and phobia. The oral antihypertensive agent nifedipine is mainly administered to patients with high systolic blood pressure (SBP) ≥160 mmHg prior to implant placement. To examine the effects of intravenous sedation and oral nifedipine on blood pressure and pulse rate in patients with perioperative high blood pressure, the clinical records of dental implant patients managed by intravenous sedation at our outpatient dental offices were retrospectively evaluated. The purpose of this clinical study is to examine whether intravenous sedation and oral administration of nifedipine is efficient for the hemodynamic for the patient with hypertension. The authors expect that it is possible not only to obtain a hemodynamic blood stable but also to prevent the medical sequelae by performing intravenous sedation and oral nifedipine for patients with hypertension.

## Methods

A retrospective review of the clinical records was conducted for 336 patients who received dental implant-related surgeries combined with intravenous sedation between January 2008 and February 2012 at our outpatient dental offices. Among the patients, 125 patients received multiple surgeries during the observation period: 4 patients underwent surgery five times, 7 patients four times, 29 patients three times, 85 patients twice, and others once. The following surgical procedures were performed in a total of 516 patients: dental implant placement (466 patients), sinus lift surgery and dental implant (28 patients), socket lift and dental implant (10 patients), and guided bone regeneration and dental implant (12 patients).

We performed surgeries after medical consultation when patients had a history of hypertension or cardiovascular or cerebrovascular diseases. History of ischemic heart disease, renal dysfunction, diabetes mellitus, cerebral infarction, or articular rheumatism was documented in 17 patients in the hypertensive group and 16 patients in the normotensive group. They were confirmed stable and well controlled for implant surgery. For patients who received therapeutic drugs, surgery was performed following daily medication.

The patients were allowed to have water or snacks until 2 h before the visit. On arrival at the office, systolic blood pressure (SBP), diastolic blood pressure (DBP), and pulse rate (PR) were measured using an automatic blood pressure monitor with an oscillometric method (HEM-1010, Omron Healthcare, Kyoto, Japan). The cephalic vein was cannulated with a 22-G disposable intravenous catheter. Nifedipine capsule (10 mg) was orally administered to patients with sustained increases in SBP ≥160 mmHg for 30 min from baseline measurement. A noninvasive blood pressure monitoring system with electrocardiogram (ECG) monitor and pulse oximeter (Moneo BP-88, Omron Healthcare, Kyoto, Japan) was mounted, and blood pressure was measured at 2- to 5-min intervals. In patients with a history of cardiovascular disease, ECG was continuously monitored. Following confirmation of a definite decline of blood pressure or SBP <160 mmHg, infiltration anesthesia and/or conduction anesthesia was administered using 1 to 3 cartridges (1.8 to 5.4 ml) of 2% lidocaine containing 1/80,000 epinephrine.

Following confirmation of a sufficient anesthetic effect, intravenous sedation with continuous infusion of propofol 1 to 2 mg/kg/h and midazolam 20 to 40 μg/kg bolus together with inhalation of oxygen 3 L/min via nasal cannula was initiated. After confirming Verrill sign, implant surgery was initiated. During operation, the propofol dose was adjusted to maintain the optimum conscious sedative condition (level 2 on the Ramsay sedation scale) [5], and local anesthesia was added when the patient complained of pain. On completion of surgery, administration of oxygen and propofol were terminated and the patient was observed for about 1 h, until normal cognitive and motor functions were restored.

The subjects were divided into two groups: a normotensive group with no history of hypertension and a hypertensive group with a history of hypertension. Thirteen patients who had no history of hypertension were included in the hypertensive group, since they indicated SBP ≥160 mmHg on arrival at the office and were later diagnosed with essential hypertension by cardiologists. Furthermore, the patients in the hypertensive group were divided into two subgroups: with or without nifedipine administration.

From the clinical chart, data of SBP, DBP, PR, and percutaneous oxygen saturation (SpO_2_) were sampled at the point of arrival to the office, prior to the initiation of sedation, 30 min after the initiation of operation, and on completion of operation. Furthermore, rate pressure product (RPP: SBP × PR) was calculated.

There were two primary outcome measures: (1) incidence of improved hypertension following oral nifedipine and (2) incidence of normal ranges of hemodynamic parameters during surgery. The secondary outcome variable was incidence of hypertension related to perioperative complications.

### Statistical analysis

In this study, we used data from all cases (516 cases) for statistical analysis.

Data were described as mean ± standard deviation. The unpaired *t* test was used to compare demographic variables between groups. Fisher's exact test was used to compare ratios of patients in hypertensive group between subgroups. One-way analysis of variance (ANOVA) followed by Tukey's multiple comparison test was performed to examine the change in the values of parameters. Repeated measures ANOVA followed by Dunnett's multiple comparison test was used to compare the values of parameters in groups at each time point. Statistical analysis was performed using Prism 5 for Windows Ver. 5.01 (GraphPad Software Inc., San Diego, CA, USA). The significance level was set at *p* < 0.05.

### Ethical approval

This study protocol was approved by the ethics committee of Japanese Dental Society of Anesthesiology (No. 2015–4).

## Results

Patient demographics and clinical characteristics are summarized in Table 1. There were significant differences in age (*p* < 0.0001) and duration of surgery (*p* = 0.025) between normotensive and hypertensive groups. On arrival at the office, values of all hemodynamic parameters, including SBP, DBP, PR, and RPP, were higher in the hypertensive group than in the normotensive group (*p* < 0.0001).

**Table 1:** **Demographic and clinical characteristics**

	**Normotensive group**	**Hypertensive group**	***p*** **value**
Number (male: female)	410 (127: 283)	106 (37: 69)	0.170
Age (year mean ± SD)	59 ± 11	65 ± 10	<0.0001
Weight (kg)	54.5 ± 9.2	57.1 ± 10.9	0.204
Values of circulation parameters on arrival at office			
SBP (mmHg)	133 ± 19	165 ± 22	<0.0001
DBP (mmHg)	76 ± 13	99 ± 20	<0.0001
PR (bpm)	79 ± 13	90 ± 19	<0.0001
RPP (bpm × mmHg)	10,574 ± 2,614	12,367 ± 5,771	<0.0001
Preoperative oral nifedipine	0	44 (42%)	<0.0001
Duration of surgery (min)	40 ± 20	35 ± 17	0.025
Duration of sedation (min)	69 ± 25	65 ± 22	0.114

SBP, systolic blood pressure; DBP, diastolic blood pressure; PR, pulse rate; RPP. rate pressure product.

Tables 2 and 3 indicate incidences of patients with SBP ≥160 mmHg and RPP ≥12,000 bpm × mmHg, and perioperative hemodynamic changes, respectively. On arrival at the office, 66 patients (62%) in the hypertensive group and 41 patients (10%) in the normotensive group revealed high SBP ≥160 mmHg. Thirty minutes later, SBP declined to less than 160 mmHg in most of the patients in the normotensive group. On the other hand, in 30 patients (33%) in the hypertensive group, high blood pressure on arrival obviously declined to around or less than 160 mmHg, while in the remaining patients in the group who showed a mean SBP of 182.1 ± 13.8 mmHg on arrival, the blood pressure did not clearly decrease after 30 min of rest. Oral nifedipine administered to patients with sustained high blood pressure decreased SBP to 144.7 ± 23.1 mmHg by 28.1 ± 9.3 min after administration, which was similar to that in hypertensive patients without nifedipine.

**Table 2:** **Incidence of high blood pressure and high rate pressure product**

	**SBP (>160 mmHg)**	**RPP (>12,000 bpm × mmHg)**
Normotensive group (*N* = 410)		
On arrival at the office	41 (10.0%)	111 (27.1%)
Prior to sedation	31 (7.6%)	72 (17.6%)
During operation	1 (0.2%)	1 (0.2%)
Completion of operation	3 (0.7%)	9 (2.2%)
Hypertensive group without preoperative oral nifedipine (*N* = 62)		
On arrival at the office	22 (35.5%)**	34 (54.8%)**
Prior to sedation	13 (21.0%)**	27 (43.5%)**
During operation	2 (3.2%)*	5 (8.1%)**
Completion of operation	5 (8.1%)**	6 (9.7%)**
Hypertensive group with preoperative oral nifedipine (*N* = 44)		
On arrival at the office	44 (100%)**	40 (90.9%)**
Prior to sedation	8 (18.2%)*	24 (54.5%)**
During operation	1 (2.2%)	4 (9.1%)**
Completion of operation	3 (6.8%)*	8 (18.2%)**

*p <0.05, **p <0.01 vs normotensive group (Fisher's exact test). SBP, systolic blood pressure; RPP, rate pressure product.

**Table 3:** **Changes in values of hemodynamic parameters**

	**SBP (mmHg)**	**DBP (mmHg)**	**PR (bpm)**	**RPP (bpm × mmHg)**
Normotensive patients (*N* = 410)				
On arrival at the office	133.0 ± 18.4	76.4 ± 12.5	79.2 ± 13.4	10,603 ± 2,623
Prior to sedation	128.5 ± 18.3*	70.5 ± 11.9*	76.8 ± 13.0*	9,927 ± 2,485*
During operation	109.7 ± 11.9*	62.7 ± 10.0*	70.6 ± 10.2*	7,768 ± 1,555*
Completion of operation	115.9 ± 14.2*	66.1 ± 11.3*	70.4 ± 10.7*	8,196 ± 1,796*
Hypertensive patients without preoperative oral nifedipine (*N* = 62)				
On arrival at the office	152.5 ± 17.6	88.3 ± 11.8	84.6 ± 14.3	12,972 ± 3,055
Prior to sedation	143.2 ± 18.1*	77.4 ± 9.6*	82.7 ± 13.4	11,874 ± 2,540*
During operation	118.9 ± 15.5*	67.2 ± 10.0*	75.2 ± 12.8*	8,977 ± 2,164*
Completion of operation	125.4 ± 19.1*	68.8 ± 12.6*	74.7 ± 10.9*	9,360 ± 1,977*
Hypertensive patients with preoperative oral nifedipine (*N* = 44)				
On arrival at the office	182.1 ± 13.8	102.8 ± 12.5	87.2 ± 17.1	15,901 ± 3,623
Prior to sedation	144.7 ± 23.1*	77.3 ± 15.9*	89.3 ± 16.1	12,986 ± 3,437*
During operation	119.8 ± 16.5*	65.8 ± 10.0*	78.0 ± 12.3*	9,413 ± 2,265*
Completion of operation	130.1 ± 18.8*	71.4 ± 13.2*	78.4 ± 13.6*	10,294 ± 2,728*

*p <0.01 vs value on arrival at the office (Dunnett's multiple comparison test). SBP, systolic blood pressure; DBP, diastolic blood pressure; PR, pulse rate; RPP, rate pressure product.

Although the mean SBP in the hypertensive group was significantly higher than that in the normotensive group during operation, SBP <160 mmHg was maintained in all patients except three in the hypertensive group (2.8%) and one in the normotensive group (0.2%). The values of DBP in patients in the hypertensive group were higher than those in the normotensive group throughout the observation course, and changes in DBP in each group were similar to those in SBP.

On arrival at the office, RPP ≥12,000 bpm × mmHg was found in 74 patients (70%) in the hypertensive group and 111 patients (27%) in the normotensive group. More than 90% of the patients with preoperative nifedipine showed high RPP on arrival at the office. Among patients with high RPP, all patients in the normotensive group, 10% of the patients without nifedipine in the hypertensive group and 35% of patients with nifedipine in the hypertensive group had RPP <12,000 bpm × mmHg until initiation of intravenous sedation. The values of RPP during operation under intravenous sedation were maintained at a normal range except in nine patients (8.5%) in the hypertensive group and one patient (0.2%) in the normotensive group.

In patients with oral nifedipine in the hypertensive group, the PR value slightly increased prior to initiation of intravenous sedation (*p* = 0.224) and then significantly decreased until completion of the operation (*p* < 0.001).

All patients stated a pleasant feeling and amnesia during surgery. No complication occurred during surgery, and no cognitive and motor dysfunctions were observed 1 h after surgery. The patients revealed SBP of >160 mmHg during and at completion of operation showed maximum SBP of 180 mmHg in the normotensive group, 190 mmHg on the hypertensive group without preoperative oral nifedipine, and 180 mmHg in the hypertensive group with preoperative oral nifedipine. They did not complaint any symptom such as headache, confusion, and chest pain. Upon leaving the office, high SBP of the patients decreased to the level on arrival without any antihypertensive treatment.

## Discussion

In 44 (8.5%) of the 516 implant surgery cases, oral nifedipine had to be administered, since preoperative SBP was higher than 160 mmHg in these patients. Within 30 min of administration of nifedipine, SBP of hypertensive patients decreased to a similar range as that of hypertensive patients who did not need administration of oral nifedipine. Intravenous sedation after nifedipine administration to hypertensive patients resulted in stable hemodynamics during implant surgery.

The Seventh Report of the Joint National Committee on Prevention, Detection, Evaluation, and Treatment of High Blood Pressure [6] classified hypertensive patients into five categories based on systolic or diastolic blood pressure. Patients with normal blood pressure (<120/80 mmHg), prehypertension (120 to 139/80 to 89 mmHg), or stage I hypertension (140 to 159/90 to 99 mmHg) can receive regular dental care, though a stress reduction protocol is necessary for stage I hypertension [2,7]. In accordance with the guidelines during oral surgery, the blood pressure of hypertensive patients should be maintained at a normal or prehypertension level. RPP is a reliable predictor of myocardial oxygen consumption [8], and RPP >12,000 bpm × mmHg is associated with myocardial ischemia [9,10]. In this study, although blood pressure was managed by a physician, hypertensive patients showed SBP >160 mmHg when they visited the dental office for dental implant surgery, and 50% of hypertensive patients showed high RPP after 30 min of rest. In patients presenting with high blood pressure and high RPP, anxiety and fear must be reduced by conscious sedation and antihypertensives to prevent cardiovascular complications during dental implant surgery.

Increases in SBP due to psychological stress are proportional to age and baseline blood pressure [4]. Intravenous sedation stabilizes measurable changes in blood pressure and pulse rate due to fear and anxiety about dental treatment and has been used to manage patients with ischemic heart disease and hypertension [11]. In this study, the effect of intravenous sedation was as follows: SBP and RPP, compared with those prior to intravenous sedation, were decreased by 15% and 20% in patients with normal blood pressure, 15% and 25% in hypertensive patients without oral nifedipine, and 15% to 20% and 20% to 30% in hypertensive patients with administered nifedipine, respectively. That is, SBP and myocardial oxygen consumption of prehypertension and stage I hypertension can be reduced to the levels recommended for dental treatment before surgery by intravenous sedation.

For patients with stage 2 hypertension before operation, it is difficult to maintain the recommended blood pressure during surgery using only intravenous sedation, and it is necessary to decrease blood pressure by antihypertensive drugs. In this study, the blood pressure of patients with sustained hypertension was reduced to stage I hypertension about 30 min after administration of oral nifedipine. On the other hand, the decrease in RPP after oral nifedipine administration was not less than 12,000 bpm × mmHg, which could be due to the fact that an increase in pulse rate with nifedipine by reflex tachycardia. Thereafter, blood pressure and RPP during surgery under intravenous sedation has remained at levels similar to those of hypertensive patients with well-controlled blood pressure. Maximum effect (21.4% decreases in SBP) appears in 30 to 60 min and lasts about 3 h on oral administration of nifedipine [12]. The half-lives of oral nifedipine, diltiazem and verapamil, and calcium antagonists are 0.2 to 1 h, 6 to 8 h, and 6 to 8 h, respectively [13]. Since oral nifedipine has the properties of fast onset (30 to 45 min) [14] and relatively short duration, it is suitable for outpatient dental implant surgery and is useful in perioperative management of patients with hypertension.

The overdose of vasoconstrictor that is added to the local anesthetic in order to prolong the anesthetic effect and hemostatic action may cause increased blood pressure and arrhythmias. Elevation of blood pressure in hypertensive patients is greater than that in normotensive patients during dental surgery [15]. Although there is an increase in blood pressure and tachycardia when using three cartridges of local anesthetic containing epinephrine 1:10,000 (5.4 ml), there are no adverse symptoms in patients with normal blood pressure [16]. Little recommended that the amount of local anesthetic solution administered should be less than two cartridges (3.6 ml) for patients with hypertension [1]. Nakamura et al. reported that patients with essential hypertension who have been administered nifedipine can receive less than 3.6 cartridges of local anesthetic containing epinephrine 1:80,000 (6.4 ml) [17]. The administration of exogenous epinephrine with local anesthesia produces the highest plasma concentration in 3 to 6 min and lasts for 20 min [18]. It has been reported that the anesthetic rate of 2% lidocaine containing 1:10,000 epinephrine is 47% 45 min after administration and 27% 60 min after administration [19]. During dental implant surgery which requires a relatively long duration and a wide field in patients with hypertension, administration of conduction anesthesia including inferior alveolar block and posterior superior alveolar nerve block is desirable. When the patient complains of pain, it is important to add local anesthesia while monitoring blood pressure to prevent increased blood pressure caused by pain.

Implant surgery is performed in patients with a wide age range, including elderly patients with hypertension. Dentists or oral surgeons often encounter hypertensive patients who are undiagnosed or noncompliant. Among Japanese over the age of 30, 60% of men and 44.6% of women suffer from high blood pressure, and 33.8% of men and 25.6% of women with a history of hypertension have not been managed medically [20]. In this study, though 13 of the patients did not have a history of hypertension, they were diagnosed with essential hypertension by a physician because they had high blood pressure before surgery. Among patients with a history of high blood pressure, 31 patients (29%) showed high blood pressure before surgery. Because there are many of dental patients with undiagnosed or noncompliant hypertension, blood pressure measurement before treatment, particularly invasive surgery, is indispensable.

For dental implant surgery in hypertensive patients who are not adequately controlled, the application of intravenous sedation and preoperative antihypertensive medication would be useful in order to prevent perioperative hypertension crisis including hypertension emergency with end-organ damage or hypertension urgency without end-organ damage. Since sublingual administration of immediate-release (IR) nifedipine may cause side effects such as significant decrease in blood pressure, reflex tachycardia, and acute myocardial infarction [21], the sublingual administration of IR nifedipine to hypertension crisis has not been approved by the Food and Drug Administration (1985) and Japanese Society of Hypertension Guidelines for the Management of Hypertension (2000). Since we could manage patients with high blood pressure without any cerebrovascular complications by oral administration of nifedipine under closely monitoring, it may be concluded that preoperative administration of oral nifedipine to patients with high blood pressure may be effective to prevent hypertensive crisis due to sudden rise in blood pressure during surgery. Further studies are necessary to evaluate the usefulness of captopril, clonidine, and labetalol, which have been reported as alternatives to nifedipine in emergency hypertension [22-24] in patients with high blood pressure.

## Conclusions

In this study, we showed that the stable hemodynamic was obtained by performing intravenous sedation and oral administration of nifedipine for patients with hypertension. It is important not only to understand the systemic management of the patient but also to obtain stabled hemodynamic by performing intravenous sedation and oral administration of nifedipine for patients with hypertension in order to perform the implant surgery safely, and it could be possible to prevent the medical sequelae.

## Abbreviations

SBP: Systolic blood pressure
DBP: Diastolic blood pressure
PR: Pulse rate
RPP: Rate pressure product
ECG: Electrocardiogram
SpO_2_: Percutaneous oxygen saturation
ANOVA: Analysis of variance
IR: Immediate-release
